# From Birth to Weaning: A Window of Opportunity for Microbiota

**DOI:** 10.3390/nu16020272

**Published:** 2024-01-17

**Authors:** Valentina Biagioli, Greta Volpedo, Antonella Riva, Paolo Mainardi, Pasquale Striano

**Affiliations:** 1 Department of Neurosciences, Rehabilitation, Ophthalmology, Genetics, Maternal and Child Health, University of Genoa, 16126 Genoa, Italy; greta.volpedo@edu.unige.it (G.V.); riva.anto94@gmail.com (A.R.); pmainardi54@gmail.com (P.M.); pstriano@unige.it (P.S.); 2 IRCCS Istituto Giannina Gaslini, 16147 Genoa, Italy

**Keywords:** microbiota, human milk oligosaccharides, complementary feeding, gut–brain axis, weaning

## Abstract

(1) Background: The first 1000 days of life constitute a critical window of opportunity for microbiota development. Nutrients play a crucial role in enriching and diversifying the microbiota, derived not only from solid food but also from maternal dietary patterns during gestation. (2) Methods: We conducted a comprehensive literature review using the PubMed database, covering eleven years (2013–2023). We included English-language reviews, original research papers, and meta-analyses, while excluding case reports and letters. (3) Results: Consensus in the literature emphasizes that our interaction with a multitude of microorganisms begins in the intrauterine environment and continues throughout our lives. The existing data suggest that early nutritional education programs, initiated during pregnancy and guiding infant diets during development, may influence the shaping of the gut microbiota, promoting long-term health. (4) Conclusions: Further research is necessary in the coming years to assess potential interventions and early nutritional models aimed at modulating the pediatric microbiota, especially in vulnerable populations such as premature newborns.

## 1. Introduction

From the first moments of life, the newborn’s body begins a mutualistic relationship with numerous microorganisms. This condition represents a true “superorganism” composed of bacteria, viruses, archaea, and unicellular eukaryotes. *Bacteroidetes* and *Firmicutes* are the major bacterial phyla, with subgroups such as *Fusobacteria, Cyanobacteria*, *Proteobacteria*, *Verrucomicrobia*, *Actinobacteria*, and a few others [1]. These microorganisms colonize our entire organism, including the integumentary system and mucosal cavities (e.g., pulmonary, oral, and vaginal); however, most are found in the gastrointestinal tract (GIT). Moreover, the colon contains more than 70% of the microorganisms colonizing the GIT [2]. The intestinal microbiota represents a “vital organ”, which, owning to the numerous connective pathways such as the neural, endocrine, immunological, and metabolic pathways, can communicate with areas that are anatomically distant from one another [3].

**Figure 1:**
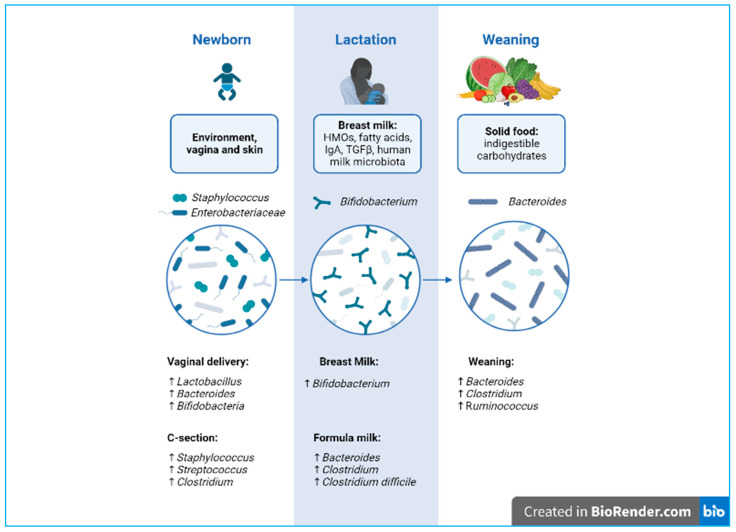
Developmental phases of microbiota biodiversity from delivery to weaning. Created with BioRender.com.

A balanced intestinal microbial ecosystem, called eubiosis, is effective in controlling a variety of infectious diseases. Therefore, balanced nutrition from qualitative and quantitative points of view and proper food processing to preserve their organoleptic and nutritional properties are fundamental for human health and for maintaining a healthy gut microbial composition [2]. On the contrary, the prolonged use of antibiotics causes an imbalance between the different strains that make up the microbiota, referred to as dysbiosis [4], a condition characterized by a shift in the composition of commensal bacterial strains toward a more pathogenic profile [2]. This change can lead to an increase in intestinal permeability, consequently triggering an inappropriate immune response [5]. This so-called “leaky gut” is often associated with conditions such as dysbiosis and gastrointestinal symptoms, which also appear to play a key role in the pathogenesis of autism spectrum disorder (ASD) [6,7]. Moreover, scientific evidence shows that microbiome dysbiosis correlates to the pathophysiology of allergies and autoimmune disorders [8]. Notably, the contribution of the human gut microbiota in early life is fundamental for the development and maturation of the infant mucosal and immune system. Furthermore, our GIT and the commensal microorganisms that inhabit it play a key role in the two-way communication system with our immune system [9]. Immediately beneath the different intestinal layers, there are a plethora of immune cells, known as gut-associated lymphoid tissues (GALT) [10]. The intestinal microbiota can also modulate the migration of neutrophils and regulate the differentiation of T cells into T helper (Th) 1, 2, and 17 cells [11,12]. Moreover, the intestinal microbiome of a newborn in the first three years of life, also due to the diversity of the nutritional substrates that reach the intestine, is distinguishable from that of an adult, as it has a lower microbial diversity index (Figure 1). A healthy intestinal microbiota remains relatively stable throughout adulthood; however, during the ageing process, it can undergo disturbances by exogenous factors (antibiotics, diet, geographical location, etc.) and endogenous factors (cellular stress, inflammatory state, pre-existing diseases, etc.) [13]. This paper reviews the impact of nutrition from pregnancy to early life in newborns and how lifestyle and diet influence microbial communities during development. 

## 2. Materials and Methods

A literature search was conducted for all articles indexed by PubMed, encompassing the time period between 2013 and 2023. The bibliographic search was conducted during the months of September and October 2023. For the search strategy, we used keywords including “Microbiota-gut-brain axis”, “Weaning”, “Complementary Feeding”, and “Human Milk Oligosaccharides”.

Studies were selected if they met the following criteria: (i) original research papers, (ii) reviews, and (iii) meta-analyses. The exclusion criteria were the following: (i) case reports, (ii) letters and commentaries, and (iii) articles that were not in English. For each keyword, according to our search criteria and with a filter applied (free full text, clinical trial, meta-analysis, in the last 10 years, species humans, and English language) for “Microbiota-gut-brain axis”, we found 79 articles; for the keyword “Weaning”, we found 117 articles; for the keyword “Complementary Feeding”, we found 8 articles; for the keyword “Human Milk Oligosaccharides”, we found 74 articles. Of these we have selected 85 articles for our manuscript.

**Figure 2:**
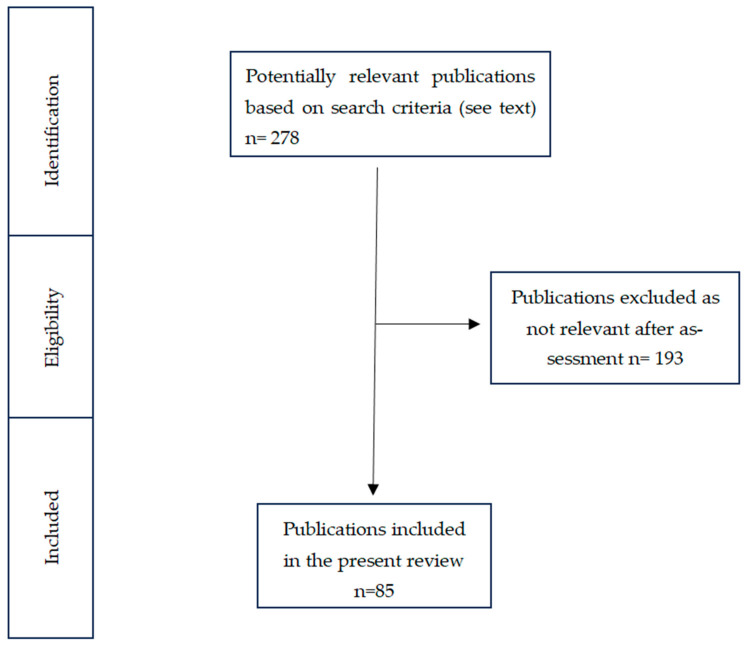
Flow diagram that guided our literature review and article selection.

Through the established search strategy, 278 articles were found, of which 85 were relevant and included in this study, while 193 were excluded. A flow diagram of the selection process is shown in Figure 2.

## 3. Results in the Literature

### Development of the Gastrointestinal Tract in Term and Preterm Infants

The human GIT begins in the mouth; extends along anatomical regions such as the esophagus, stomach, small intestine, colon, and rectum; and ends in the anus. Including the intestine, villous structures, and microvilli, the estimated surface area in the adult is 200 m^2^ [14]. Our intestinal microbiota resides along this entire surface and plays a role of primary importance in the absorption, digestion, and metabolism of nutrients [15]. Furthermore, the gut flora produces metabolites with paracrine and endocrine properties, establishing communication networks that, if in balance, can promote the general health of the individual or induce illness [16].

The maturation of the human GIT is a crucial part of human development. In fact, intestinal development in newborns proceeds in parallel with brain development and immune maturation [17].

To adequately absorb and digest the first nutrients (colostrum and breast milk), newborns need their GIT to be adequately developed, both structurally and functionally, to perform adequate peristalsis, continence of the tone of the gastroesophageal sphincter, and coordination in sucking–swallowing [18]. Premature infants, ≤28 weeks old, can experience nutritional emergency due to a highly underdeveloped and immature GIT [19]. Moreover, 50% of preterm newborns experience abdominal distention, with delayed gastric emptying, slowed intestinal peristalsis, and a consequent delay in the passage of meconium [20]. Because of this, most preterm newborns need to be admitted to a neonatal intensive care unit for stabilization and treatment. Prolonged hospitalization involves exposure to nosocomial pathogens [18], increasing the newborn’s risk of infections. To prevent this, premature babies are often treated with broad-spectrum antibiotics during their hospital stay, which may play a role in the pathogenesis of necrotizing enterocolitis (NEC) [21]. NEC is a gastrointestinal complication of prematurity and is characterized by severe inflammation and bacterial translocation, resulting in the formation of intraluminal cells, air, and intestinal perforation in the most severe cases [19]. This disorder can lead to an increased risk of infections, both acute and long-term, associated with an increased risk of developing immunological conditions as well as metabolic disorders [22]. Finally, these factors can lead to reduced microbial diversity and health in newborns [23].

## 4. Discussion

### 4.1. What Is the “Window of Opportunity”?

#### 4.1.1. Maternal Nutrition and Infant Gut Microbiome: How Materno–fetal Exchange Influences the Development of Neonates

Pregnancy is a unique biological process that involves numerous physiological changes to support the health of both the mother and the fetus. Moreover, the placenta is a highly specialized organ, which divides the fetal and maternal environments. Historically, the placenta was considered a sterile organ, but, in recent years, new evidence has demonstrated the possible existence of a placental microbiome. In 2014, the first studies based on bacterial DNA demonstrated the existence of a placental microbiota even during full-term and normal pregnancies [24].

Metagenomics and metabolomics studies have shown that the maternal intestinal microbiota plays an important role in the transport of metabolites derived from commensal bacteria across the placental barrier and is therefore able to modulate fetal development [25]. Exogenous factors such as diet and drug intake, as well as endogenous factors such as maternal stress and alterations in the metabolic state, influence the maternal intestinal microbiota [26]. For example, treatment with broad-spectrum antibiotics during gestation has been associated with an early onset of inflammatory bowel disease (IBD) in the child [27] and reduced microbiota diversity in the mother [28]. Moreover, high-birth-weight infants (>3.6 kg) with mothers who are obese during gestation have a greater risk of attention deficit disorders [29]. Children of overweight or obese mothers also have a greater risk of preterm birth (<37 weeks), with a consequent risk of pediatric neurological disorders [30]. Furthermore, gestational diabetes mellitus, a condition that occurs in 5% of pregnancies, is associated with dysbiosis of both the maternal and neonatal intestinal microbiota [29].

Even during a normal pregnancy, the maternal microbiota changes its composition. For example, *Faecalibacterium* is a producer of short-chain fatty acids (SCFAs), which tends to decrease in the last trimester of pregnancy [30]. SCFAs such as acetate, propionate, and butyrate are of fundamental importance as they provide 60–70% of the energy needs of colonocytes [31]; in particular, butyrate promotes cellular repair and regeneration processes, maintaining epithelial integrity [32,33]. For these reasons, paying attention to the diet of pregnant women is essential to support changes in the intestinal microbiota in this critical phase.

The fetal microbiota composition is therefore strongly influenced by prenatal factors and strictly dependent on maternal lifestyle and habits. For example, smoking and a Western diet style rich in sugars, processed foods, and saturated fats negatively influence the fetal microbiota [34].

The intrauterine life and the first years of life represent a critical time to shape the newborn’s microbiota. In humans, the composition of the intestinal microbiota appears to stabilize around 2.5–4 years of life [35]. Before this stabilization occurs, the intestinal microbiota of the newborn appears to be much more susceptible to stimuli that modulate it [36,37].

#### 4.1.2. Vaginal Delivery and Vertical Transmission of Microbiota

During natural birth, the fetus passes through the maternal vaginal canal, coming into contact with the microbial ecosystem present (vaginal and fecal). This has a long-term influence on the composition and development of the newborn’s microbiota [38].

#### 4.1.3. Caesarean Section

During a cesarean (C-) section, the baby has increased contact with the mother’s integumentary system and with the microbes present in the hospital environment [39]. Contrary to vaginal births, C-section-delivered newborns show reduced microbiota diversity and richness, with significantly lower levels of bacterial genera such as *Bifidobacterium*, *Lactobacillus*, and *Bacteroides*, as well as higher levels of *Staphylococcus*. Interestingly, clinical studies highlight a relationship between C-section and immune disorders, such as allergies and asthma [40]. However, further studies are needed to clarify the long-term role of the altered microbiota.

## 5. Human Milk and Neonatal Brain Development

Human colostrum (HC), the first form of breast milk, activates the post-natal maturation processes that make the newborn nutritionally independent from the mother, especially in cases such as preterm infants. In the weeks following birth, premature newborns are highly susceptible to systemic infections, brain damage, and a compromised GIT [41]. The progress made with nutritional and immunological approaches has significantly improved the management and care strategies for premature infants. In particular, the role of HC is fundamental, and its composition changes according to the newborn’s needs [42]. For example, the HC of mothers with preterm babies has a higher protein content than that of mothers who delivered at term to keep up with the infant’s demanding nutritional needs. This protein-rich HC has been considered the best way to prevent NEC in preterm babies [43], possibly due to the high levels of protective factors such as lactoferrin [44,45,46], which are also important in reducing late-onset sepsis in preterm babies [47].

Furthermore, HC is rich in immune factors that can protect the newborn from infectious diseases, while also providing growth factors for the development of the newborn’s GIT. Due to the action of specific proteins, HC also stimulates the newborn’s immune system [42]. However, if the newborn is very preterm or has a low birth weight, breast milk is not sufficient to support the infant’s development. HC can be enriched with specific nutrients to guarantee a sufficient intake of energy and micronutrients in the newborn.

The nutrients in breast milk, however, are strongly influenced by maternal characteristics such as nutrition, mental health, and lifestyle choices. Breastfeeding is the gold standard for infant nutrition, as it adequately provides not only the nutrients that the newborn needs but also different microbiota populations [48]. For these reasons, breastfeeding is highly recommended for up to 6 months of life.

### 5.1. Macro- and Micronutrient Composition

**Figure 3:**
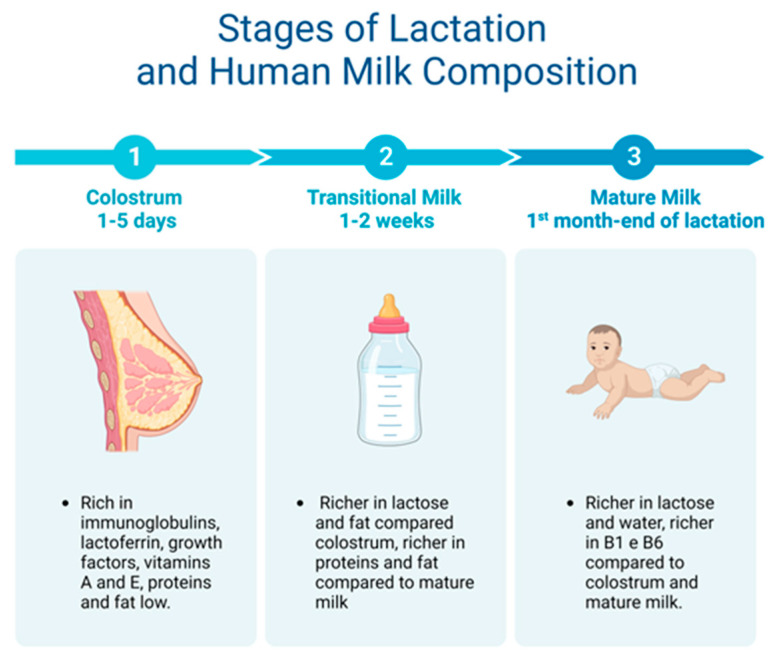
From colostrum to mature milk and their different properties.

Breast milk is made up of macro- and micronutrients specialized in supporting the physiological and metabolic functions, neural development, and the intestinal microbiota of the newborn [49]. Two weeks after giving birth, human milk is considered fully mature. Mature milk contains 6.9–7.2% carbohydrates, 3–5% fats, 0.8–0.9% proteins, and 0.2% mineral components [44] (Table 1; Figure 3).

### 5.2. Human Milk Oligosaccharides

Human milk oligosaccharides (HMOs) are complex, indigestible glycans present in high quantities in breast milk [50]. HMOs act as real prebiotics as they cross the intestinal lumen and are fermented by the intestinal microbiota [51]. After lactose and lipids, HMOs are the third most abundant components of human milk, with high quantities (20–25 ng/L) in HC, gradually decreasing to 5–15 ng/L in mature milk.

HMOs prevent infection with pathogenic bacteria such as *Salmonella*, *Campylobacter*, and *Listeria* and promote *Bifidobacterium* [52], thus limiting the growth of bacteria potentially harmful to the newborn [48].

### 5.3. Other Bioactive Compounds

Some bioactive components of breast milk such as insulin-like growth factors (IGFs) 1 and 2 act as energy substrates for newborns, promoting the growth and development of various tissues. Among the bioactive components, there is lactoferrin, a glycoprotein that binds iron and has antimicrobial activity. Furthermore, high levels of lactoferrin are reported in HC, while there is a gradual decline in its levels to mature milk.

Furthermore, secretory (S)IgA and SIgG are the most abundant immunoglobulins in milk, providing support to the newborn’s immune functions [53] by preventing the adhesion of pathogens to the surface of epithelial cells. SIgA is present at concentrations up to 12 mg/L in HC.

Other bioactive nutrients include milk fat globule membranes (MFGMs), which are secreted by breast lactocytes and have a complex structure made up of glycolipids, phospholipids, carbohydrates, and proteins. The composition of MFGMs varies at different stages of lactation and is influenced by exogenous maternal factors such as diet and environment. MFGMs such as mucin-1 and lactadherin have antimicrobial effects, promoting the growth of commensals and disfavoring the establishment of pathogens in the neonatal intestine [54]. Furthermore, sphingomyelin and phosphatidylcholine are components present in MFGMs. Choline is a common precursor of the neurotransmitter acetylcholine, which is involved in central nervous system functions, such as memory and attention. In particular, sphingomyelins are important for myelination [55].

### 5.4. Breast Milk Microbiome

Initially, human breast milk (HBM) was considered a sterile fluid, and the presence of bacteria was considered to be contamination or infection [44]. To date, scientific evidence has detected microbial metabolites in HC [56]. This discovery has led to growing interest in the HMB microbiome.

After the vaginal birth canal, the HBM is the second source of microbes for the newborn. Breastfed babies have been predicted to consume up to 8 × 10^5^ bacteria each day. There are various mechanisms proposed to explain the transmission of the microbiota through breastfeeding, including contamination from the mother’s integumentary system and retrograde flow from the newborn oral cavity to the ductal tissue [44]. In support of these hypotheses, notable similarities have been found between the adult skin microbiome and the milk microbiome, in particular the presence of *Corynebacterium* and *Staphylococcus*. Furthermore, the microbes present in breast milk (such as *Bifidobacterium* and *Lactobacillus*) are not present in the neonatal oral cavity before the initiation of breastfeeding. Some species, such as *Bifidobacterium* and *Lactobacillus*, are shared in both the mother’s HBM and the infant’s stool, highlighting that HBM contributes to the vertical transmission of commensal bacteria. Interestingly, *Lactobacillus* and *Bifidobacterium* are also the strains most commonly used as probiotics. In particular, *Lactobacillus* spp. have inhibitory actions against pathogenic bacteria, such as *Shigella* spp., *Salmonella* spp., and *E. coli*, preventing their adhesion in the intestinal lumen [53].

## 6. Infant Formula

### 6.1. Macronutrients in Infant Formula

Even though breastfeeding is the first choice for the health of the newborn, many factors can influence the decision, such as medical, occupational, or family support problems and complications. When breastfeeding is not an option, infant formula (IF) becomes part of the newborn’s nutrition.

Most of the constituent proteins in IF derive from bovine milk, which has fewer essential amino acids than HBM. The protein content of IFs of bovine origin varies between 2 and 3 g/100 mL, higher than the protein level present in HBM (1.4–1.6 g/100 mL in HC and 0.8–1.0 g/mL in mature milk). This excess of proteins and amino acids overstimulates the β-pancreatic cells and is strongly associated with early adiposity and a greater risk of becoming overweight in adulthood [57]. Furthermore, caseins are also present in greater quantities in bovine milk than in HBM, resulting in difficulty in digestion. Furthermore, IF contains varying quantities of carbohydrates and bioactive components such as HMOs, which have a very important prebiotic role, favoring commensal microbial strains [58].

### 6.2. Supplements in Infant Formula: Probiotics, Prebiotics, and Postbiotics

In recent years, research has increasingly focused on the effect of prebiotics and probiotics on the infant microbiota. These studies, combined with a growing interest from industries in IF, have led to the continuous improvement in IF to create a formulation that resembles HBM as much as possible. New IFs have currently been developed with enrichment in bioactive ingredients such as probiotics, prebiotics, and postbiotics.

In October 2013, the International Scientific Association for Probiotics and Prebiotics (ISAPP) re-examined the concept of probiotics, redefining them as “live microorganisms that, when administered in adequate amounts, confer a health benefit on the host”. This definition is inclusive of a wide range of microbes and therapeutic applications. The seven most commonly used genera in commercial products are *Bifidobacterium*, *Saccharomyces*, *Streptococcus*, *Escherichia*, *Lactobacillus*, *Enterococcus*, and *Bacillus* [59]. Prebiotics, on the other hand, are food substrates that, once ingested, are selectively fermented by certain microbial strains, conferring benefits to the host. The most commonly used prebiotics include galactooligosaccharides (GOS), fructooligosaccharides (FOS), and inulin. Finally, postbiotics are a preparation of inanimate microorganisms with immunomodulatory, anti-inflammatory, trophic, and antimicrobial effects, in a preclinical model [60].

The supplementation of IFs with probiotics is safe for infants and children [61], with the aim of modulating the activity of the newborn’s intestinal microbiota, promoting eubiosis [62] with a bifidogenic effect [63]. Moreover, formulas supplemented with FOS and GOS result in significant reductions in the prevalence of childhood asthma and eczema [63]. Finally, postbiotics, such as SCFAs, improve insulin sensitivity and glucose tolerance [64]; furthermore, some microorganisms can produce and modulate various neuroactive compounds. For example, some strains of *Lactobacillus* and *Bifidobacterium* can produce gamma-aminobutyric acid (GABA), which is the dominant inhibitory neurotransmitter in the brain. Other bacterial species such as *Enterococcus* and *Escherichia coli* can produce serotonin. Lastly, some species of bacilli can produce dopamine neurotransmitters [65].

### 6.3. Infant Formula, Risks, and Future Directions

The use of IF stimulates glucose metabolism, increases insulin sensitivity, and causes early advanced adiposity that persists into adulthood. However, further studies are needed to delve into the safe dosages and beneficial effects in pediatric disorders and premature infants to create more standardized protocols for this age group [66].

## 7. Dietary Nutrients Shape the Gut Microbiota: From Infancy to Childhood

### 7.1. Microbiota Maturation during Weaning

Malnourished and under- or over-nourished infants and children develop an immature intestinal microbiota, which can compromise child growth [67].

The transition to solid foods implies an increase in the intake of proteins, carbohydrates, lipids, and fibers, which leads to an increase in microbial richness and diversity. For example, a randomized control trial demonstrated that the introduction of solid foods (including meat, cereals, and fruit) led to an increase in the diversity of the gut microbiota [68].

During this transition, the count of milk-related bacteria, such as *Bifidobacterium*, *Lactobacillaceae*, and *Enterobacteriaceae*, decreases, while bacteria such as *Bacteroides*, *Akkermansia*, and *Ruminococcaceae*, which are capable of fermenting and digesting the more complex nutrients, are introduced during weaning and expand [69].

An excessive delay in the colonization of the intestinal microbiota even once weaning has begun may derive from contexts of food insecurity, malnutrition, and infections, with a negative impact on the health and nutritional status of the child [70,71]. However, further studies are needed to more thoroughly understand microbial changes during weaning and their long-term effects in humans.

### 7.2. Establishment of Nutritional Habits from Solid Food Introduction

It is known that the mother’s eating habits and qualitative choice of nutrients during pregnancy are essential for the development of the newborn’s taste and olfactory preferences [72]. The olfactory system, which includes the olfactory bulbs of the brain that are already functioning at the 24th week of gestation, develops in parallel with that of taste: swallowing and breathing the amniotic fluid offers the fetus the flavors and odors of the food consumed by the mother [73].

Bad eating habits, excessive protein intake, and a sedentary lifestyle are among the most important risk factors for the development of obesity both in childhood and during adult life [74,75]. In this context, complementary nutrition becomes a unique opportunity to establish correct eating habits from an early age and to start education on taste, with the involvement of all family members [76]. This evidence underlines the importance of the family environment in the child’s nutritional education [77].

### 7.3. Nutrient–Microbiota Interactions and Brain Development during Early Life

Microbial signals are crucial not only for intestinal eubiosis but also for the healthy and correct development of neuronal circuits in the brain [78]. Intestinal microbes communicate with the central nervous system (CNS), secreting signaling molecules that move through the circulatory system and crossing the intestinal epithelium and the blood–brain barrier (BBB) [79,80]. Brain development begins during fetal life and continues through adolescence through critical processes such as neurogenesis, synaptogenesis, and myelination [81]. During childhood, neurons are highly plastic in response to environmental stimuli, and the intestinal microbiota has a role in the structural and functional aspects of brain development and maturation [82]. Moreover, studies conducted on germ-free mice have shown that the absence of gut microbiota is accompanied by cognitive impairments, such as memory dysfunction, anxiety, and abnormal motor activity [83].

## 8. Conclusions

Starting from intrauterine life, the gut microbiota composition and biodiversity are influenced by environmental factors and nutrition. Beyond the different dietary regimes, the vastness and biodiversity of the microbial world that accompanies us throughout our lives cannot be addressed with a “one-size-fits-all” approach. The period of gestation and the birth of the newborn represents a window of opportunity to modulate the health status of the newborn through noninvasive and inexpensive methods such as education on healthy nutrition for microbial biodiversity. The goal of future nutritional approaches should be to develop personalized diets following microbiota analysis to modulate and restore a healthy intestinal microbiota, thus laying the foundations for a personalized and preventative medical approach aimed at the maintenance of an individual’s health status and not just the treatment of disease.

## Figures and Tables

**Table 1:** Macronutrients in human milk (these values may vary if the newborn is preterm) [44].

	Colostrum (Day 0–5)	Mature Milk (Day 14 and Later)
Energy kcal/100 mL	50–60	65–70
Carbohydrates g/L	50–62	60–70
Total Protein g/L	14–16	8–10
Total Fat g/L	15–20	35–40
